# Rapid water transfer from extracellular to intracellular spaces following oral ingestion

**DOI:** 10.1016/j.jphyss.2026.100088

**Published:** 2026-07-10

**Authors:** Hiroyuki Terawaki, Masahiko Y. Kida

**Affiliations:** a Clinical Laboratory Department, St. Luke’s International University, Tokyo, Japan; b Division of Advanced Community-Based Care for Lifestyle-Related Diseases, Fukushima Medical University, Fukushima, Japan; c Internal Medicine, Fukushima Jukokai Hospital, Fukushima, Japan

**Keywords:** Oral ingestion, Water transfer, Impedance method, Insulin

## Abstract

Research on the effects of oral ingestion on extracellular and intracellular water balance is scarce. To measure the postprandial change in extracellular–intracellular water balance, 28 healthy adults (mean age 39 years, 20 women, 8 men) were recruited, and their extracellular–intracellular water distribution was measured at five time points: immediately before lunch (fasting), immediately after lunch (postprandial), and 30, 60, and 120 min after lunch using a BCM® body composition measuring device (Fresenius Medical Care, Germany). The results did not show significant changes in the total water volume, whereas the extracellular–intracellular distribution ratio decreased significantly from 0.7468 ± 0.0496 during fasting to 0.7376 ± 0.0473 during postprandial periods, which gradually increased over the next 120 min (0.7491 ± 0.0569). The estimated mean postprandial loss of extracellular fluid was 132 mL. Overall, oral ingestion promotes the transient transfer of body water from extracellular to intracellular spaces.

## Background

Oral ingestion influences vital signs, such as blood pressure and pulse rate. In healthy individuals, oral ingestion increases pulse rate [1] and cardiac output [2], [3], [4]: At this time, blood pressure remains stable or increases slightly [5]. Conversely, in people with Parkinson’s disease [6] or in the older population [7], a condition known as “postprandial hypotension” is frequently observed, in which oral ingestion conversely causes a decrease in blood pressure. Changes in blood flow distribution are considered the mechanism by which oral ingestion alters vital signs: an adequate autonomic response maintains blood pressure, whereas an insufficient response decreases blood pressure [3], [8], [9].

Oral ingestion can influence blood fluid dynamics by altering blood flow distribution [10] and, in addition, the extracellular/intracellular distribution (E-I) ratio. The practical application of bioimpedance analysis (BIA) has allowed accurate and noninvasive assessment of body fluid volume, including the E-I ratio. Understanding the physiological changes caused by oral ingestion on the body fluid volume and the E-I ratio can be useful in planning therapeutic strategies for postprandial hypotension.

This study aimed to investigate the effect of oral ingestion on the body fluid volume, including the E-I ratio, in 28 healthy participants using BIA. Consequently, an immediate postprandial shift in body fluids from extracellular to intracellular spaces was found. To our best knowledge, this is the first report to examine the effects of standard oral ingestion on extracellular and intracellular water balance.

## Methods

### Participants and study protocol

Twenty-eight healthy volunteers (mean age, 39 years; 20 women, 8 men; mean body mass index [BMI] 22.3 kg/m^2^) working at Fukushima Jukokai Hospital were recruited for this study. Participants’ profiles are summarized in Table 1.

**Table 1:** Participants' profile.

Factor	Group	
n		28
Sex (%)	Female	20 (71.4)
	Male	8 (28.6)
Age (years)		39 (30−46)
Fasting body mass index (kg/m^2^)		22.3 (20.2–24.0)
Height (cm)		159.0 (153.8–168.5)

**Fig. 1:**
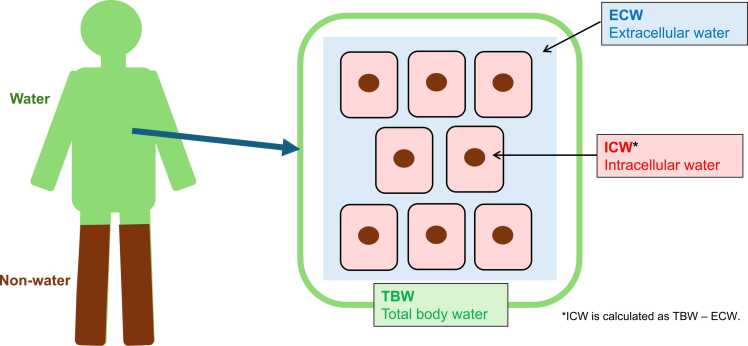
Relationship between extracellular water, intracellular water, and total body water.

BIA assessment was performed using a BCM® body composition monitor (Fresenius Medical Care, Bad Homburg, Germany), which provides fluid-related measurements of total body water (TBW, L), extracellular water (ECW, L), and intracellular water (ICW, L), which is the difference between TBW and ECW (Fig. 1). The E-I ratio was calculated as ECW/ICW. BIA assessment also provides body composition-related measurements, such as lean tissue mass (LTM, kg) and overhydration (OH, L), which represents the estimated excess body water beyond the minimum extracellular fluid volume required to maintain proper circulation. BCM provided all parameters (TBW, ECW, ICW, LTM, and OH) as numerical values. The precision of BIA assessment using a BCM body composition monitor was confirmed previously [11].

**Fig. 2:**
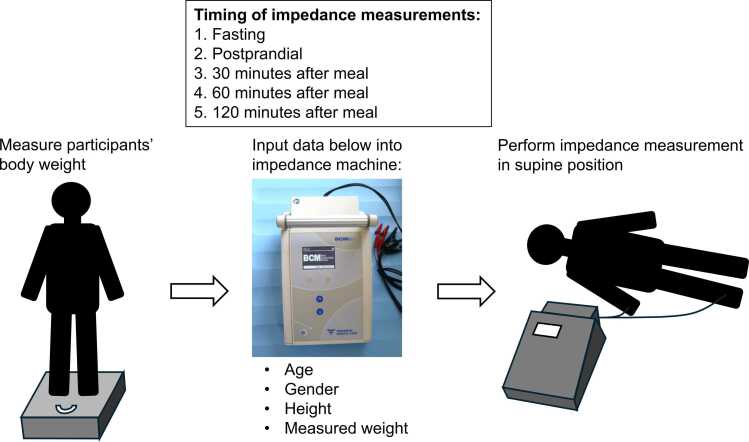
Measurement protocol of this study.

BIA measurements were performed at five time points: immediately before lunch (fasting), immediately after lunch (postprandial), and 30, 60, and 120 min after lunch, as well as immediately after weighing (Fig. 2). All participants consumed breakfast at home and were allowed to drink fluids until the end of the body composition assessment to avoid dehydration, which can lead to unstable measurements [12]. However, snacks, sweets, and beverages containing sugar and/or milk were prohibited. Lunch was served at 12:00 PM and consisted of a standard hospital meal (calories, 500–600 kcal; protein, 20–24 g; salt, approximately 2 g). Urination and defecation were not prohibited until 120 min after lunch.

### Statistical analysis

All statistical analyses were performed using EZR version 1.33 (Saitama Medical Center, Jichi Medical University, Saitama, Japan), which is a graphical user interface for R (R Foundation for Statistical Computing, Vienna, Austria). More precisely, it is a modified version of the R Commander designed to add statistical functions frequently used in biostatistics [13].

Numerical data are presented as the mean ± standard deviation (normally distributed data) or median with interquartile range (non-normally distributed data), and categorical data as numbers and percentages. The significance of differences between paired data between two (namely, between fasting and postprandial) was assessed using paired *t*-test, and those between more than three using univariate type III repeated-measures analysis of variance. Pearson’s correlation coefficient was used to evaluate the relationship between two variables. Differences were considered statistically significant at *p* < 0.05.

## Results

**Fig. 3:**
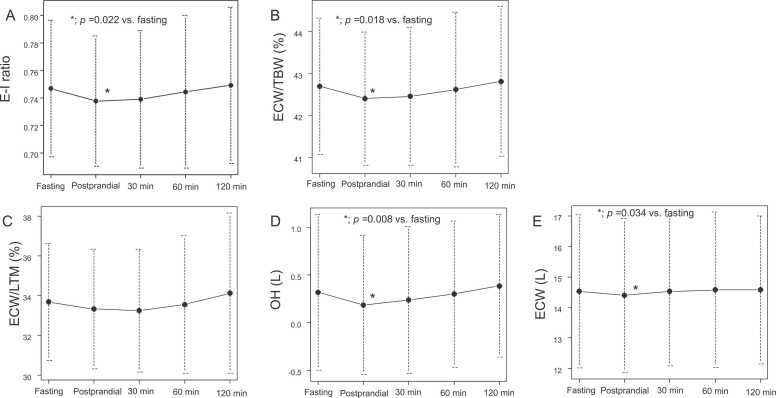
**Chronological change in the E-I ratio (A), ECW/TBW (B), ECW/LTM (C), OH (D), and ECW (E).** E-I ratio, extracellular/intracellular distribution; ECW, extracellular water; LTM, lean tissue mass; OH, overhydration; TBW, total body water.

The measurement results are summarized in Table 2. TBW and LTM did not show significant changes throughout the study period from before lunch to 120 min after it. However, as shown in Fig. 3A, the mean E-I ratio decreased significantly from 0.7468 during fasting to 0.7376 during postprandial periods and 0.7389 30 min after meals and gradually increased over the next 120 min (0.7491). Likewise, the variables with ECW as the numerator, namely, ECW/TBW and ECW (Figs. 3B and 3E), as well as the variable OH reflecting ECW (Fig. 3D), showed similar fluctuations over time as the E-I ratio, suggesting rapid water transfer from extracellular to intracellular spaces following oral ingestion. The difference between fasting and postprandial OH (ΔOH), i.e., the estimated amount of water transferred from extracellular to intracellular spaces by oral ingestion, was 132 ± 244 mL (minimum, –300 mL; median, 150 mL; maximum, 700 mL). ΔOH did not show a significant relationship with age, BMI, and sex (Table 3).

**Table 2:** Results of bioimpedence analysis.

Variable		Mean ± SD	*p*-value
TBW (kg)	Fasting	34.11 ± 6.31	0.56
	Postprandial	34.02 ± 6.34	
	30 min	34.27 ± 6.09	
	60 min	34.34 ± 6.65	
	120 min	34.17 ± 6.29	
ICW (kg)	Fasting	19.58 ± 3.88	0.63
	Postprandial	19.63 ± 3.91	
	30 min	19.76 ± 3.75	
	60 min	19.76 ± 4.19	
	120 min	19.64 ± 4.01	
ECW (kg)	Fasting	14.53 ± 2.51	0.031*
	Postprandial	14.39 ± 2.53	
	30 min	14.52 ± 2.45	
	60 min	14.57 ± 2.55	
	120 min	14.58 ± 2.43	
LTM (kg)	Fasting	43.59 ± 9.30	0.56
	Postprandial	43.68 ± 9.41	
	30 min	44.14 ± 9.04	
	60 min	44.14 ± 10.26	
	120 min	43.68 ± 9.72	
E-I ratio	Fasting	0.7468 ± 0.0496	0.022*
	Postprandial	0.7376 ± 0.0473	
	30 min	0.7389 ± 0.0500	
	60 min	0.7443 ± 0.0554	
	120 min	0.7491 ± 0.0569	
ECW/TBW (%)	Fasting	42.70 ± 1.61	0.015*
	Postprandial	42.39 ± 1.59	
	30 min	42.46 ± 1.64	
	60 min	42.63 ± 1.85	
	120 min	42.83 ± 1.79	
ECW/LTM (%)	Fasting	33.68 ± 2.94	0.034*
	Postprandial	33.33 ± 3.01	
	30 min	33.25 ± 3.09	
	60 min	33.56 ± 3.46	
	120 min	34.12 ± 4.03	
OH (L)	Fasting	0.32 ± 0.82	< 0.001*
	Postprandial	0.19 ± 0.73	
	30 min	0.24 ± 0.77	
	60 min	0.30 ± 0.77	
	120 min	0.39 ± 0.74	

ECW; extracellular water, E-I ratio; extracellular/intracellular distribution ratio, ICW; intracellular water, LTM; lean tissue mass, OH; overhydration, TBW; total body water.

* *p* < 0.05 (univariate type III repeated-measures analysis of variance).

**Table 3:** The relationship between ΔOH and age, fasting body mass index, and sex.

	R (95% confidence interval)	*p*-value
Age (years)	−0.003 (−0.012 ─ 0.006)	0.53
Fasting body mass index (kg/m^2^)	−0.018 (−0.047 ─ 0.012)	0.22
Sex (male=1)	0.016 (−0.200 ─ 0.233)	0.88

R; Pearson’s correlation coefficient.

## Discussion

In this study, the body composition was evaluated before and after meals using BIA to examine whether oral ingestion can induce changes in ECW and ICW distribution. Consequently, this study revealed rapid (extremely short period before and after eating) water transfer from extracellular to intracellular spaces following oral intake, to the best of our knowledge, for the first time.

**Fig. 4:**
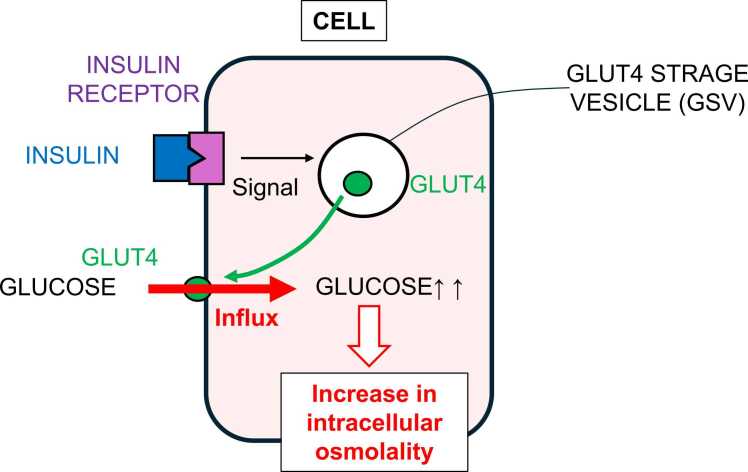
**Suspected mechanism of how oral ingestion induces body water shift from extracellular to intracellular spaces via insulin action.** Under conditions of sufficient circulating insulin and glucose, the binding of insulin to its receptor on cell membranes initiates a multistep intracellular signaling cascade that promotes the fusion and exocytosis of glucose transporter type 4 (GLUT4) storage vesicles (GSVs) with the plasma membrane, thus facilitating glucose uptake through surface-localized GLUT4, resulting in an increase in intracellular osmolality that induces a body water shift from extracellular to intracellular fractions.

The movement of water into cells after food intake, as confirmed in this study, can be caused by solutes that increase the osmotic pressure, including sodium and glucose. Sodium absorption associated with dietary intake leads to an increase in extracellular fluid volume corresponding to the amount absorbed [14], while simultaneous glucose absorption leads to an increase in extracellular fluid volume through its transfer into cells themselves and to an increase in glycogen levels in liver and muscle cells [15], [16], [17]. Insulin, which is involved in the transport of glucose into cells during feeding, contributes to the latter process [18]: Insulin moves insulin-dependent glucose transporters (GLUT4) to the cell surface, allowing water to flow into insulin-sensitive cells such as myocytes and adipocytes, and contributing to a further increase in intracellular water content (Fig. 4). Such combined effects of insulin (opening the door of cell membrane) and glucose (pouring-in through the door of cell membrane) can lead to the phenomenon confirmed in this research. Although the methodology used in this study does not clarify which organ cells receive extracellular fluid after meals, considering that the majority of intracellular fluid is found in the liver and skeletal muscle [19], and that GLUT4 is present on the surface of muscle cells, it is likely that the organ that plays the main role in absorbing 132 mL (mean value) of extracellular fluid is skeletal muscle.

Taking into the glucose-insulin action as stated above, previous reports that the viscosity-increasing agent (guar gum) [20] or the alpha-glucosidase inhibitor acarbose [21] reduces postprandial hypotension to some extent can be understood as a mechanism for its effectiveness in preventing rapid glucose absorption from the intestinal tract. Furthermore, our hypothesis that intracellular water movement is induced by glucose-insulin action via GLUT4 is consistent with the report by Unrein et al. that sugar-free amino acid beverages did not induce any water movement from extracellular to intracellular spaces [22].

The decrease in extracellular fluid observed in this study (mean, 132 mL; minimum, –300 mL; median, 150 mL; maximum, 700 mL) is far less than the amount that shifts from the whole body to the internal organs during ingestion (approximately 500 mL) [10], and appears to be well compensated for by the sympathetic response that includes vasocontriction and increased cardiac output. However, unlike changes in blood distribution associated with feeding, which are simply changes in the distribution of blood volume in veins (volumetric vessels), intracellular water shifts reduce extracellular fluid volume just like bleeding outside the body, and thus it could contribute to postprandial hypotension among participants with impaired sympathetic nervous and/or cardiac function. While, it should be noted that the amount of intracellular water movement measured in this study represents a change calculated using BIA, not an absolute value but an estimated value.

In this study, it should be noted that the meal was consumed at lunchtime, since circadian rhythms influence fluid and electrolyte regulation and cardiovascular functions, including sodium and water handling, autonomic nervous system activity, and vascular tone [23]. In other words, physiological responses to food intake can differ between lunch and feeding at different times, that is, breakfast or dinner.

This study had several limitations. First, the number of participants was too small for a stratified analysis. Second, this study lacks data on the change in vital signs including blood pressure and pulse rate. Third, the participants’ plasma insulin concentration was not measured. Fourth, all participants were Japanese; therefore, other ethnicities were not evaluated. Fifth, all participants were healthy, excluding participants with physical impairments, such as older adults and patients with autonomic disorders. Finally, this study is single-armed, lacking negative controls using calorie-free beverages or saline solutions and/or positive controls using isocaloric isotonic nutrient solutions: This nature reduces the plausibility of this study. Further studies are required to overcome these limitations.

## Conclusions

The analysis of BIA measurements in healthy participants revealed that oral ingestion promoted the transitional transfer of body water from extracellular to intracellular spaces, to our knowledge, for the first time.

## CRediT authorship contribution statement

**Hiroyuki Terawaki:** Writing – review & editing, Writing – original draft, Visualization, Validation, Methodology, Investigation, Formal analysis, Data curation, Conceptualization. **Masahiko Y. Kida:** Writing – review & editing, Visualization, Supervision, Resources, Project administration, Data curation, Conceptualization.

## Ethics approval

This study was approved by the Institutional Review Board of Fukushima Jukokai Hospital (Approval No. 00011) according to the principles of the Declaration of Helsinki. This study was presented as a wall poster to visitors of Fukushima Jukokai Hospital, and written informed consent was obtained from all participants.

## Consent for publication

Not applicable.

## Funding

The authors received no specific funding for this work.

## Declaration of Competing Interest

The authors declare that they have no known competing financial interests or personal relationships that could have appeared to influence the work reported in this paper.
